# Diphenylpyrenylamine-functionalized polypeptides: secondary structures, aggregation-induced emission, and carbon nanotube dispersibility†

**DOI:** 10.1039/c8ra02369g

**Published:** 2018-04-23

**Authors:** Ahmed F. M. EL-Mahdy, Shiao-Wei Kuo

**Affiliations:** Department of Materials and Optoelectronic Science, National Sun Yat-Sen University Kaohsiung 80424 Taiwan kuosw@faculty.nsysu.edu.tw; Chemistry Department, Faculty of Science, Assiut University Assiut 71516 Egypt

## Abstract

In this study we prepared—through ring-opening polymerization of γ-benzyl-l-glutamate *N*-carboxyanhydride (BLG-NCA) initiated by *N*,*N-*di(4-aminophenyl)-1-aminopyrene (pyrene-DPA-2NH_2_)—poly(γ-benzyl-l-glutamate) (PBLG) polymers with various degrees of polymerization (DP), each featuring a di(4-aminophenyl)pyrenylamine (DPA) luminophore on the main backbone. The secondary structures of these pyrene-DPA-PBLG polypeptides were investigated using Fourier transform infrared spectroscopy and wide-angle X-ray diffraction, revealing that the polypeptides with DPs of less than 19 were mixtures of α-helical and β-sheet conformations, whereas the α-helical structures were preferred for longer chains. Interestingly, pyrene-DPA-2NH_2_ exhibited weak photoluminescence (PL), yet the emission of the pyrene-DPA-PBLG polypeptides was 16-fold stronger, suggesting that attaching PBLG chains to pyrene-DPA-2NH_2_ turned on a radiative pathway for the non-fluorescent molecule. Furthermore, pyrene-DPA-2NH_2_ exhibited aggregation-caused quenching; in contrast, after incorporation into the PBLG segments with rigid-rod conformations, the resulting pyrene-DPA-PBLG polypeptides displayed aggregation-induced emission. Transmission electron microscopy revealed that mixing these polypeptides with multiwalled carbon nanotubes (MWCNTs) in DMF led to the formation of extremely dispersible pyrene-DPA-PBLG/MWCNT composites. The fabrication of MWCNT composites with such biocompatible polymers should lead to bio-inspired carbon nanostructures with useful biomedical applications.

## Abstract 2

PBLG chains to pyrene-DPA-2NH_2_ turned on a radiative pathway for the non-fluorescent molecule and TEM revealed these polypeptides with carbon nanotube to form PBLG/MWCNT composite.

## Graphical abstract

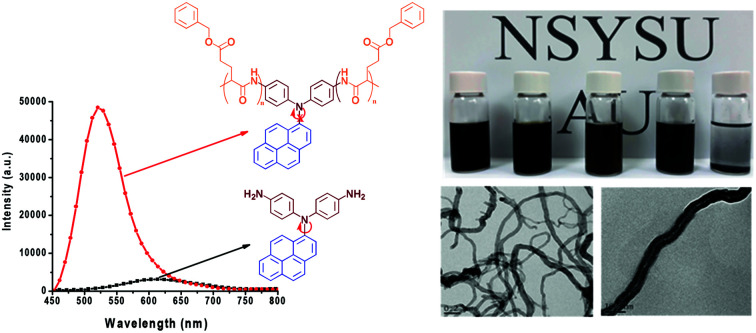

## Introduction

Polypeptides are protein-like polymers composed of repeating α-amino acid residues connected through peptide bonds (–CONH–); they are readily degraded into the corresponding α-amino acids in the human body. Polypeptides are attracting much attention for biomedical applications because of their excellent biodegradability and good biocompatibility *in vivo*;^1^ for example, they may be used as gene vectors,^2^ drug carriers,^3^ and tissue engineering materials.^4^ Notably, polypeptides can form stable hierarchically ordered structures, including rigid-rod-like α-helical and β-sheet conformations, both in solution and in the solid state.^1,5^ The α-helical and β-sheet conformations are stabilized primarily through intramolecular and intermolecular hydrogen bonds, respectively.^6^ Among the polypeptides, poly(γ-benzyl-l-glutamate) (PBLG) has been widely investigated as a synthetic polypeptide that degrades *in vivo* to l-glutamic acid, one of the essential amino acids for the human body. PBLG can be synthesized through ring-opening polymerization (ROP) of γ-benzyl-l-glutamate *N*-carboxyanhydride (BLG-NCA) or multilateral peptide monomers.^5^ Attractive properties can result when PBLG polypeptides are conjugated with other functional peptide monomers.^7^ The continued design of new supramolecular polypeptides will inevitably lead to a diverse range of prospective applications.

Carbon nanotubes (CNTs) are unique one-dimensional (1D) structures that are composed essentially of sheets of carbon atoms that are arranged in hexagons and rolled into tubes. They can be categorized in two basic forms: single-wall carbon nanotubes (SWCNTs), which feature a single roll of hexagonal carbon atoms, and multiwall carbon nanotubes (MWCNTs), which are single tubes masked into wider tubes, which are also encased into other tubes. As a result of their π-electron systems, CNTs have unique mechanical, optoelectronic, and thermal properties.^8^ Nanocomposites containing CNTs are receiving increasing attention for their potential applications in electronic devices,^9^ high-performance composites,^10^ sensors,^11^ and biological materials.^12^ Considerable efforts have been devoted to developing covalent and noncovalent techniques for controlling the aggregation of CNTs and improving their dispersion in various nanocomposites.^13^ For example, CNTs covalently modified with polypeptides on their surfaces have been examined for their biological applications.^14^ Yao *et al.* used the “graft-from” approach to synthesize polypeptide-modified MWCNTs (PBLG-MWCNT) through ROP of amino-functional MWCNTs with the BLG-NCA monomer.^15^ In addition, the synthesis of polypeptide-modified SWCNTs (PBLG-SWCNT) has been performed through the addition of azido-terminated PBLGs to SWCNTs.^16^ Nevertheless, such covalent modification can change the hybridization of the CNT's carbon atoms from sp^2^ to sp^3^, possibly weakening the mechanical, electronic, or optical properties.^17^ In contrast, noncovalent modification of CNTs involves the physical absorption of small surfactants or conjugated polymers to the CNT surface. This approach can enhance the dispersion of CNTs, while maintaining the mechanical and optoelectronic properties. The most common noncovalent modifications involve mussel inspired surface and pyrene modifications. In the former modification, MWCNTs with polydopamine (PDA) coating have been prepared *via* mussel inspired chemistry. The dopamine molecules are firstly self-polymerized into PDA under weakly alkaline aqueous conditions and then the formed PDA strongly adhered and coated on MWCNTs surface. After that the PDA coating can be further reacted with thiol, amino and acrylamide, acrylate and thiocarbonylthio-modified polymers through Michael addition reaction,^18^ atom transfer radical polymerization (ATRP),^19^ single-electron transfer living radical polymerization (SET-LRP),^20^ and reversible addition–fragmentation chain transfer polymerization (RAFT)^21^ to form surface-modified MWCNTs. However, this modification needs many steps, long reaction times. In the later modification, pyrene units are interacted strongly with the surface of CNTs through π-stacking, thereby producing homogenously dispersed pyrene/CNT composites.^22^ This method has many further advantages include short reaction time, high yield, and operation simplicity. Highly dispersed polypeptide/CNT composites are of interest for their biophysical and biomedical applications.

Pyrene is a fluorogenic unit displaying variable photophysical properties, making it useful as a fluorophore for labeling in probes for nucleic acids^23^ and metal ions.^24^ The attractive properties of pyrene include its appearance of delayed fluorescence, ready functionalization, high propensity for forming excimers, and distinct solvatochromic phenomena. In recent years, a great number of pyrene derivatives have been prepared, including oligothiophenes with pyrenyl side/end groups,^25^ tetraphenylpyrene,^26^ hexapyrenylbenzene,^27^ ethynylene-conjugated pyrene,^28^ and dipyrenebenzenes,^27^ in addition to fluorene-pyrene, carbazole-pyrene, and fluorene-carbazole-pyrene systems.^29,30^ Furthermore, polypyrenes,^31^ pyrene-starbursts,^32^ and pyrene-dendrimers^33^ have been investigated for organic electronic applications (*e.g.*, organic light-emitting devices) that take advantage of their emissive properties. Moreover, alkyl pyrenes have been incorporated as photophysical probes into synthetic peptide skeletons; their photofluorescence properties have been applied to investigate peptide conformations^34^ and the tertiary and quaternary structures of peptides.^35^ The structures and aggregation behavior of peptides containing pyrene units can be examined by monitoring the changes in their emission spectra. Although pyrene is a blue-emitting chromophore, it emits weakly when aggregated or at high concentrations, due to strong intermolecular π-stacking of these planar molecules; this phenomenon is known as aggregation-caused quenching (ACQ).^36^ The aggregation of pyrene-containing peptides leads to changes in their emission spectra—namely, decreases in the molecular extinction coefficients and red-shifts of the absorption maxima.^37,38^ Therefore, it would be interesting to discover new fluorescent pyrene-containing peptides that emit blue light in the aggregation state, as well as in the solution, with high efficient quantum yield.

Triarylamines have been studied extensively for their high charge mobilities and excellent photonic and electronic properties, and have been applied as building blocks in the construction of light-emitters, hole-transporters, and photo-conductors.^39^ Among the developed triarylamines, triphenylamine (TPA) derivatives are fluorescent in solution, but are less emissive when aggregated in the solid state; therefore, they are ACQ chromophores.^40,41^ For example, tetraphenylbiphenyl-4,4′-diamine (TPA-dimer) exhibits a strong emission in tetrahydrofuran (THF), but the emission intensity is 5.5-fold lower in the solid state. Recently, we reported the incorporation of TPA into polytyrosine (PTyr) through the ROP of the NCA amino acid at room temperature. The emission intensity of TPA itself decreased upon increasing its concentration in THF—typical ACQ behavior. In contrast, the TPA emission transformed from ACQ to aggregation-induced emission (AIE)^42,43^ after the incorporation of TPA into the main chain of polytyrosine.^44^

**Scheme 1:**
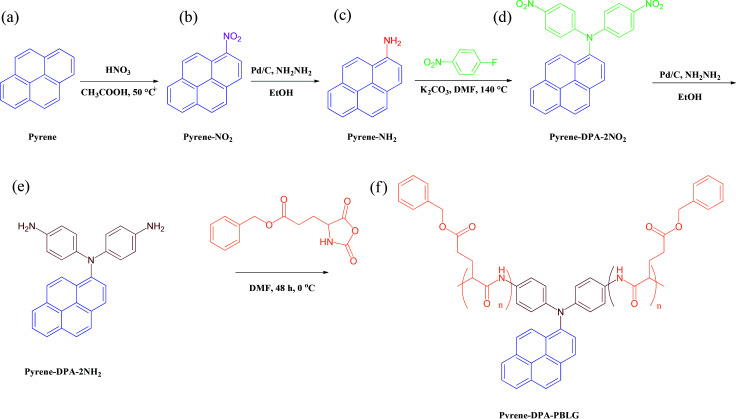
Preparation of (e) pyrene-DPA-2NH_2_ and (f) pyrene-DPA-PBLG from (a) pyrene, (b) pyrene-NO_2_, (c) pyrene-NH_2_, and (d) pyrene-DPA-2NO_2_.

To the best of our knowledge, di(4-aminophenyl)pyrenylamine, which combines the structural features of pyrene and TPA, has never previously been incorporated into polypeptide main chains, despite the interesting properties of pyrene and TPA units. Herein, we report the use of *N*,*N*-di(4-aminophenyl)-1-aminopyrene (pyrene-DPA-2NH_2_) as an initiator for the synthesis of a series of pyrene-DPA-PBLG polypeptides formed through ROP of the BLG-NCA monomer (Scheme 1). We characterized the resulting polypeptides using Fourier transform infrared (FTIR) spectroscopy, nuclear magnetic resonance (NMR) spectroscopy, and mass-analyzed laser desorption/ionization time-of-flight (MALDI-TOF) mass spectrometry. We studied of the secondary structures of these polypeptides using FTIR spectroscopy and wide-angle X-ray diffraction (WAXD). We also applied photoluminescence spectroscopy to investigate the fluorescence properties of pyrene-DPA-2NH_2_ and pyrene-DPA-PBLG. Moreover, we used transmission electron microscopy (TEM) and fluorescence spectroscopy to investigate the interactions and dispersibility of our new polypeptides when complexed with multiwalled carbon nanotubes (MWCNTs).

## Experimental section

### Materials

Pyrene and γ-benzyl-l-glutamic acid ester were used as received from Acros. Palladium on carbon (Pd/C, 10 wt%), 4-fluoronitrobenzene, hydrazine hydrate (98%), triphosgene, and potassium carbonate were obtained from Alfa Aesar. Glacial acetic acid was purchased from Fluka. Ethanol (EtOH), dimethylsulfoxide (DMSO), methanol (MeOH), THF, and acetonitrile were obtained from Merck and distilled over CaH_2_ prior to use. MWCNTs [diameters (main range): 40–60 nm; lengths: 5–15 μm] were ordered from Centron Biochemistry Technology (Taiwan). MWCNTs were purified through sonication in toluene for 1 h, ultrafiltration, and drying under vacuum overnight. BLG-NCA^45^ and 1-aminopyrene^46^ were synthesized using previously reported methods.

### *N*,*N*-Di(4-nitrophenyl)-1-aminopyrene (pyrene-DPA-2NO_2_)

In a 100 mL two-neck round-bottomed flask equipped with a stirring bar, a solution of 1-aminopyrene (1.5 g, 7.0 mmol) and K_2_CO_3_ (3.8 g, 28 mmol) in DMSO (30 mL) was stirred under N_2_ atmosphere for 10 min. 4-Fluoronitrobenzene (1.5 mL, 14 mmol) was added and then the mixture was heated at 140 °C for 24 h under a N_2_ atmosphere. The cooled mixture was poured into MeOH (200 mL) slowly; the precipitated product was filtered off and washed thoroughly with MeOH and hot water. The crude product was recrystallized (DMF/MeOH) to afford brown needles (2.7 g, 85%); mp: 271–273 °C. FTIR (KBr, cm^−1^): 1302, 1579 (NO_2_ stretch). ^1^H NMR (500 MHz, DMSO-*d*_6_) [Scheme S1(a)†]: 7.34 (d, 4H, H_j_), 7.94 (d, H_a_), 8.03 (d, H_d_), 8.13 (t, H_f_), 8.14 (d, H_k_), 8.18 (d, H_b_), 8.29 (d, H_i_), 8.30 (d, H_h_), 8.32 (d, H_e_), 8.40 (d, H_g_), 8.46 (d, H_c_). ^13^C NMR (125 MHz, DMSO-*d*_6_): 121.92 (C_2_), 121.99 (C_18_), 124.22 (C_4_), 125.96 (C_14_), 126.18 (C_19_), 126.52 (C_8_), 126.81 (C_10_), 127.35 (C_5_), 127.54 (C_9_), 127.68 (C_13_), 128.22 (C_15_), 128.41 (C_6_), 128.83 (C_12_), 130.03 (C_3_), 130.81 (C_16_), 131.10 (C_11_), 131.37 (C_7_), 137.51 (C_1_), 142.35 (C_20_), 152.42 (C_17_).

### *N*,*N*-Di(4-aminophenyl)-1-aminopyrene (pyrene-DPA-2NH_2_)

In a 100 mL two-neck round-bottomed flask equipped with a stirring bar, pyrene-DPA-2NO_2_ (2.0 g, 4.4 mmol) and 10% Pd/C (0.10 g) were suspended in EtOH (60 mL) and THF (10 mL) under a N_2_ atmosphere. The suspension was heated at 90 °C for 15 min before hydrazine monohydrate (6.5 mL) was added slowly. The mixture was stirred at 90 °C for 36 h and then it was filtered to remove the Pd/C. The filtrate was cooled, giving yellow crystals, which were filtered off and dried under vacuum at 70 °C (1.35 g, 75%); mp: 227–229 °C (DSC). FTIR (KBr, cm^−1^): 3206–3387 (N–H stretch). ^1^H NMR (500 MHz, DMSO-*d*_6_) [Scheme S1(b)†]: 4.81 (s, 4H, NH_2_), 6.47 (d, 4H, H_k_), 6.66 (d, 4H, H_j_), 7.61 (d, H_a_), 7.95 (d, H_d_), 7.99 (t, H_f_), 8.04 (d, H_i_), 8.09 (d, H_h_), 8.11 (dd, 2H, H_c_ + H_e_), 8.16 (d, H_b_), 8.19 (d, H_g_). ^13^C NMR (125 MHz, DMSO-*d*_6_): 114.88 (C_19_), 123.70 (C_4_), 123.81 (C_18_), 124.26 (C_8_+C_5_), 124.37 (C_14_), 124.99 (C_10_), 125.24 (C_2_), 125.66 (C_15_), 125.68 (C_13_), 126.06 (C_3_), 126.21 (C_9_), 126.41 (C_6_), 126.85 (C_16_), 127.82 (C_12_), 131.13 (C_11_), 131.54 (C_7_), 139.97 (C_17_), 143.77 (C_1_), 144.25 (C_20_).

### Pyrene-DPA-PBLG

BLG-NCA (1.0 g, 3.8 mmol) was weighed in a dry-box under N_2_, transferred to a three-neck round-bottom flask, and then dissolved in dry DMF (20 mL). The solution was stirred at 0 °C for 15 min prior to the introduction of a solution of pyrene-DPA-2NH_2_ (various ratios) in DMF (3 mL) using a N_2_-purged syringe. After stirring at 0 °C for 72 h, the mixture was poured into diethyl ether (Et_2_O). The precipitate was purified three times through dissolution into MeOH and reprecipitation from Et_2_O, giving a pale-yellow powder that was dried under vacuum at 40 °C overnight. Pyrene-DPA-PBLG(24): *T*_g_ = 24.2 °C; FTIR (KBr, cm^−1^): 3304, 3062, 2959, 1736, 1653, 1548, 1449, 1163, 746, 698, 610 [Fig. S1(A)†]; ^1^H NMR (500 MHz, DMSO-*d*_6_): 8.20–8.00 (br, pyrene), 7.56 (br, 4H, H_g_), 7.30 (br, 10H, H_e_), 6.56 (br, 4H, H_f_), 5.05 (br, 4H, H_d_), 4.01 (br, 2H, H_c_), 2.49 (br, 4H, H_b_), 2.19–1.88 (br, 4H, H_a_) [Fig. S2(A)†]. Pyrene-DPA-PBLG(19): *T*_g_ = 25.4 °C; FTIR (KBr, cm^−1^): 3299, 3053, 2952, 1736, 1649, 1545, 1445, 1168, 742, 696, 606 [Fig. S1(B)†]; ^1^H NMR (500 MHz, DMSO-*d*_6_): 8.19–8.00 (br, pyrene), 7.60 (br, 4H, H_g_), 7.32 (br, 10H, H_e_), 6.58 (br, 4H, H_f_), 5.05 (br, 4H, H_d_), 4.06 (br, 2H, H_c_), 2.49 (br, 4H, H_b_), 2.15–1.88 (br, 4H, H_a_) [Fig. S2(B)†]. Pyrene-DPA-PBLG(9): *T*_g_ = 23.6 °C; FTIR (KBr, cm^−1^): 3295, 3061, 2952, 1728, 1655, 1548, 1455, 1162, 744, 694, 612 [Fig. S1(C)†]; ^1^H NMR (500 MHz, DMSO-*d*_6_): 8.20–8.00 (br, pyrene), 7.60 (br, 4H, H_g_), 7.25 (br, 10H, H_e_), 6.52 (br, 4H, H_f_), 5.08 (br, 4H, H_d_), 4.05 (br, 2H, H_c_), 2.48 (br, 4H, H_b_), 2.18–1.88 (br, 4H, H_a_) [Fig. S2(C)†]. Pyrene-DPA-PBLG(6): *T*_g_ = 23.1 °C; FTIR (KBr, cm^−1^): 3295, 3062, 2946, 1736, 1653, 1545, 1453, 1163, 740, 694, 608 [Fig. S1(D)†]; ^1^H NMR (500 MHz, DMSO-*d*_6_): 8.16–8.00 (br, pyrene), 7.75 (br, 4H, H_g_), 7.36 (br, 10H, H_e_), 6.61 (br, 4H, H_f_), 5.04 (br, 4H, H_d_), 4.01 (br, 2H, H_c_), 2.36 (br, 4H, H_b_), 2.22–1.80 (br, 4H, H_a_) [Fig. S2(D)†].

### Dispersion of MWCNTs and pyrene-DPA-PBLG

MWCNTs were dispersed into DMF (5 mL) through sonication for 2 h. A solution of pyrene-DPA-PBLG in DMF (1 mL) was then added dropwise into the MWCNT dispersion. The mixture was sonicated for 2 h and then stirred at room temperature for 24 h. The mixture was centrifuged (5000 rpm, 60 min) and then the supernatant was subjected to ultrafiltration through PALL disc membrane filters (FP-450 PVDF filters) to give MWCNT/pyrene-DPA-PBLG composites. These composites were redispersed in various solvents through sonication for 2 min.

### Characterization

FTIR spectra were measured using a Bruker Tensor 27 FTIR spectrometer; samples were prepared using the KBr disk method; 64 scans were collected at room temperature at a spectral resolution of 4 cm^−1^; the sample films were suitably thin to obey the Beer–Lambert law. ^1^H and ^13^C NMR spectra were recorded using an Agilent VMRS-600 NMR spectrometer at 600 and 150 MHz, respectively; CDCl_3_ and DMSO-*d*_6_ were used as solvents, and tetramethylsilane (TMS) as the external standard. The molecular weights of the synthesized polypeptides were obtained from MALDI-TOF mass spectra, recorded using a Bruker Daltonics Autoflex III spectrometer (operating parameters: ion source 1, 19.06 kV; ion source 2, 16.61 kV; lens, 8.78 kV; reflector 1, 21.08 kV; reflector 2, 9.73 kV). Differential scanning calorimetry (DSC) and thermogravimetric analysis (TGA) were performed under a N_2_ atmosphere using Q-20 and Q-50 thermogravimetric analyzers (TAs), respectively; for DSC, the samples were places in a sealed aluminum pan and heated from 40 to 200 °C (heating rate: 10 °C min^−1^); for TGA, the samples were heated from 30 to 800 °C (heating rate: 20 °C min^−1^). WAXD patterns were measured using the wiggler beam line BL17A1 of the National Synchrotron Radiation Research Center (NSRRC), Taiwan; a triangular bent Si (111) single crystal was used to obtain a monochromatic beam having a wavelength (*λ*) of 1.24 Å; the samples were annealed at 180 °C for 2 h, and then cooled to room temperature, prior to measurement. UV-Vis absorption spectra were recorded using an Ocean Optics DT 1000 CE 376 spectrophotometer. PL spectra were recorded using a LabGuide X350 fluorescence spectrometer, with a 450 W Xe lamp as the continuous light source; a small quartz cell (dimensions: 0.2 × 1.0 × 4.5 cm^3^) was used to adjust the solution sample. TEM images for the samples were recorded using a JEOL-2100 transmission electron microscope operated at an accelerating voltage of 200 kV.

## Results and discussion

### Synthesis of pyrene-DPA-2NH_2_

**Fig. 1:**
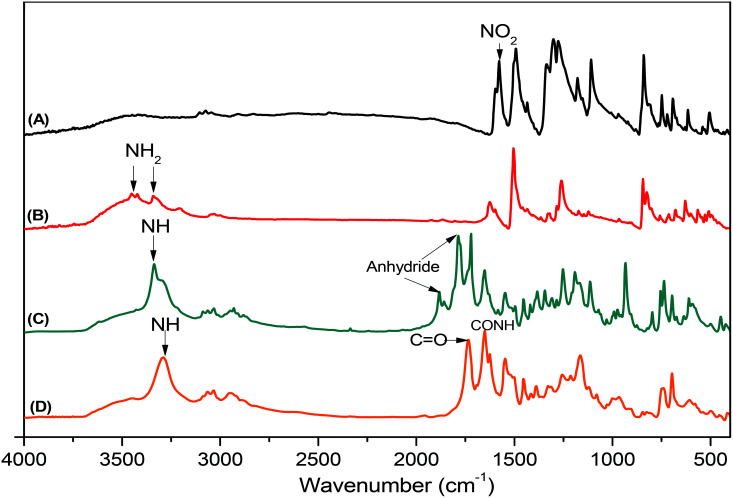
FTIR spectra of (A) pyrene-DPA-2NO_2_, (B) pyrene-DPA-2NH_2_, (C) BLG-NCA, and (D) pyrene-DPA-PBLG(6), recorded at room temperature.

**Fig. 2:**
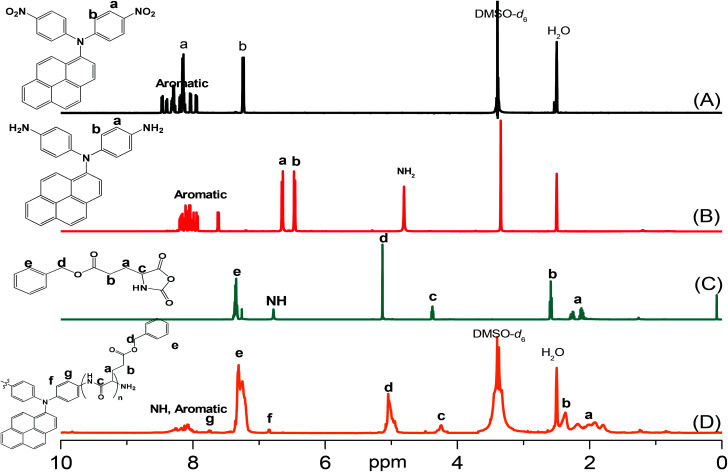
^1^H NMR spectra of (A) pyrene-DPA-2NO_2_, (B) pyrene-DPA-2NH_2_, (C) BLG-NCA, and (D) pyrene-DPA-PBLG(6).

**Fig. 3:**
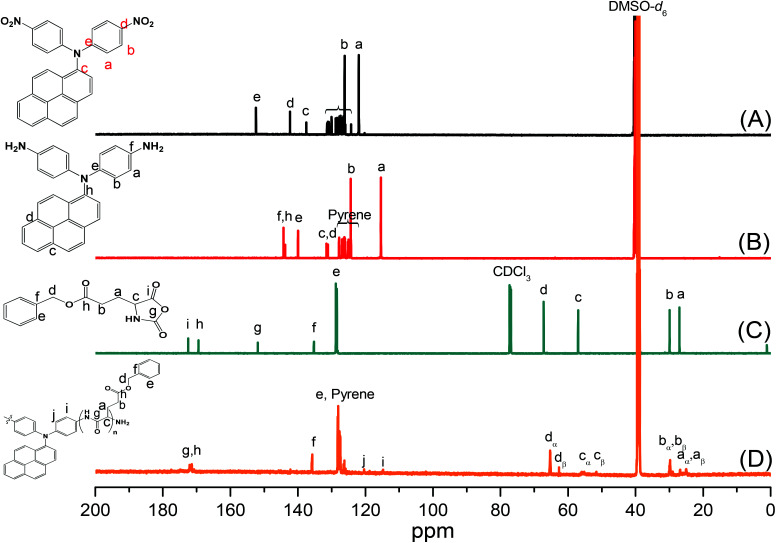
^13^C NMR spectra of (A) pyrene-DPA-2NO_2_, (B) pyrene-DPA-2NH_2_, (C) BLG-NCA, and (D) pyrene-DPA-PBLG(6).

Pyrene-DPA-2NH_2_, used as a monomer initiator for the ROP, was synthesized according to the synthetic strategy outlined in Scheme 1. First, the dinitro-compound (pyrene-DPA-2NO_2_) was prepared through *N*,*N*-diarylation of 1-aminopyrene with 4-fluoronitrobenzene in the presence of K_2_CO_3_ at 140 °C in DMSO. Reduction of pyrene-DPA-2NO_2_ with hydrazine hydrate in the presence of a catalytic amount of 10% Pd/C in EtOH at 90 °C provided the diamine-initiator compound pyrene-DPA-2NH_2_. FTIR and ^1^H and ^13^C NMR spectroscopic analyses confirmed the chemical structures of pyrene-DPA-2NO_2_ and pyrene-DPA-2NH_2_. Fig. 1(A) and (B) present the FTIR spectra of pyrene-DPA-2NO_2_ and pyrene-DPA-2NH_2_, respectively. The spectrum of pyrene-DPA-2NO_2_ exhibits two sharp signals at 1302 and 1579 cm^−1^ for the NO_2_ group, while the spectrum of pyrene-DPA-2NH_2_ exhibits three sharp signals at 3206, 3302, and 3387 cm^−1^ for symmetric and asymmetric NH stretching. Fig. 2(A) and (B) present the ^1^H NMR spectra of pyrene-DPA-2NO_2_ and pyrene-DPA-2NH_2_, respectively. The spectrum of the dinitro compound features characteristic signals for nitro-substituted aromatic rings at 7.37 and 8.30 ppm, with signals for the pyrene protons in the range 7.94–8.46 ppm. The spectrum of the diamine featured a broad signal at 4.81 ppm, attributed to the NH_2_ groups, in addition to signals for the aromatic protons. The ^13^C NMR spectrum of pyrene-DPA-2NO_2_ features [Fig. 3(A)] a signal for the C–NO_2_ unit at 142.35 ppm; for pyrene-DPA-2NH_2_ [Fig. 3(B)], the signal for the C–NH_2_ unit appears at 144.25 ppm. These features are all consistent with the high-yield synthesis of the diamine-functionalized initiator pyrene-DPA-2NH_2_.

### Synthesis of pyrene-DPA-PBLG

As illustrated in Scheme 1, pyrene-DPA-PBLG polypeptides, with two PBLG chains linked to a pyrene-DPA unit, were synthesized at room temperature through ROP of the BLG-NCA monomer initiated by the diamine pyrene-DPA-2NH_2_. The successful formation of these polypeptides was elucidated from their FTIR, ^1^H and ^13^C NMR, and MALDI-TOF mass spectra. The FTIR spectrum of BLG-NCA [Fig. 1(C)] features two signals at 1787 and 1883 cm^−1^, attributed to the two modes of anhydride C=O stretching, in addition to a signal for the ester at 1718 cm^−1^. After polymerization of BLG-NCA [Fig. 1(D) and S1†], the two anhydride peaks disappeared and a new absorption appeared at 3299 cm^−1^ for NH stretching. In addition, absorption peaks emerged for the side chain ester, amide I, and amide II groups of pyrene-DPA-PBLG at 1728–1736, 1649–1653, and 1545–1548 cm^−1^, respectively. Fig. 2(C) presents the ^1^H NMR spectrum of BLG-NCA. The signals of the NH group and methylene protons (H_d_) appeared at 6.78 and 5.13 ppm, respectively. The formation of pyrene-DPA-PBLG was confirmed from its ^1^H NMR spectrum [Fig. 2(D) and S2†]: the aromatic protons (H_a_, H_b_) of pyrene-DPA-2NH_2_ (at 6.47 and 6.66 ppm) were shifted downfield (to 6.56–6.61 and 7.56–7.75 ppm) after formation of the PBLG chains. Moreover, signals for the pyrene moiety and methylene protons (H_d_) were present in the spectrum of pyrene-DPA-PBLG. Fig. 3(C) displays the ^13^C NMR spectrum of the BLG-NCA monomer; signals for the three C=O groups (C_h_, C_i_, C_g_) and three CH_2_ groups (C_a_, C_b_, C_d_) appeared at 169.50, 172.56, 151.80, 26.90, 29.92, and 67.31 ppm, respectively. In addition, the signal of the amino acid α-carbon atoms (C_c_) appeared at 56.90 ppm. Fig. 3(D) presents the ^13^C NMR spectrum of pyrene-DPA-PBLG(6); the signals of the two anhydride C=O groups and the ester C=O group were absent, but two new signals appeared at 171.45 and 171.77 ppm for the ester C=O and amide carbon atoms. The signals at 65.22 and 55.69 ppm represent the methylene carbon atom (C_d_) and the amino acid α-carbon atom (C_c_) of the α-helical conformation, respectively.

**Fig. 4:**
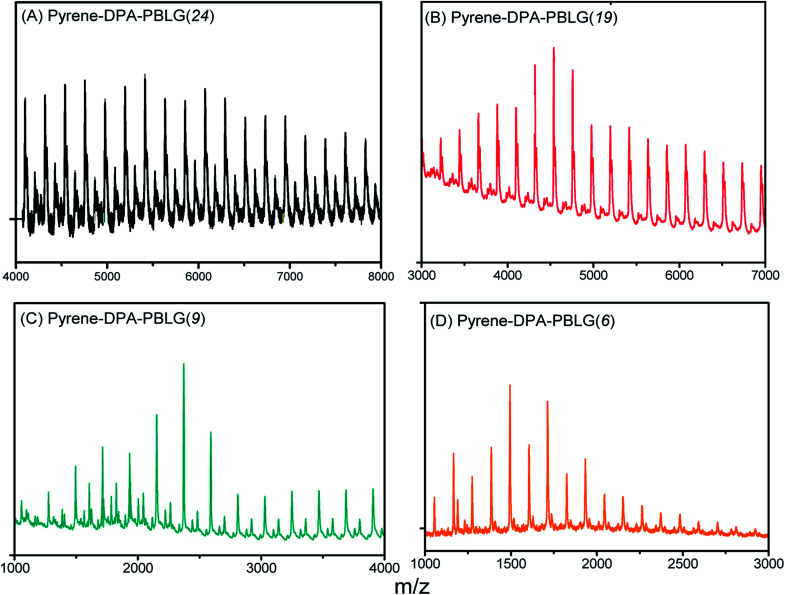
MALDI-TOF mass spectra of (A) pyrene-DPA-PBLG(24), (B) pyrene-DPA-PBLG(19), (C) pyrene-DPA-PBLG(9), and (D) pyrene-DPA-PBLG(6).

We calculated the molecular weights of the polypeptides pyrene-DPA-PBLG from their ^1^H NMR and MALDI-TOF mass spectra. Taking pyrene-DPA-PBLG(9) as an example, the integration ratio between the aromatic protons H_f_ and the methylene protons H_d_ provided a number-average molecular weight of 2370 g mol^−1^ (Table 1), in good accordance with the value (2367 g mol^−1^) calculated from the MALDI-TOF mass spectrum [Fig. 4(C)]. As indicated in Fig. 4 and Table 1, the molecular weights of all the pyrene-DPA-PBLG polypeptides, determined from the ^1^H NMR and MALDI-TOF mass spectra, correlated well. Moreover, the mass difference between pairs of adjacent peaks in all of the MALDI-TOF spectra of the synthesized polypeptides was *m*/*z* 219, consistent with a BLG repeating unit. Taken together, our results from FTIR, ^1^H and ^13^C NMR, and MALDI-TOF spectroscopy confirmed the successful preparation of the pyrene/TPA fluorophores-containing pyrene-DPA-PBLG polypeptides; Table 1 summarizes the results.

**Table 1:** Molecular weight values of polypeptides from ^1^H-NMR and MALDI-TOF mass spectra^a^

Polypeptide	From ^1^H-NMR	From MALDI-TOF
Pyrene-DPA-PBLG(24)	*M*_n_ = 5766 (*n* = 24)	*M*_n_ = 5411 (*n* = 23)
		*M*_w_ = 5632, PDI = 1.04
Pyrene-DPA-PBLG(19)	*M*_n_ = 4565 (*n* = 19)	*M*_n_ = 4543 (*n* = 19)
		*M*_w_ = 4759, PDI = 1.05
Pyrene-DPA-PBLG(9)	*M*_n_ = 2370 (*n* = 9)	*M*_n_ = 2367 (*n* = 9)
		*M*_w_ = 2587, PDI = 1.09
Pyrene-DPA-PBLG(6)	*M*_n_ = 1713 (*n* = 6)	*M*_n_ = 1491 (*n* = 5)
		*M*_w_ = 1601, PDI = 1.07

a *n* refers to the total number of incorporated units in the two PBLG chains per pyrene-DPA-PBLG.

### Secondary structures of pyrene-DPA-PBLG polypeptides

**Fig. 5:**
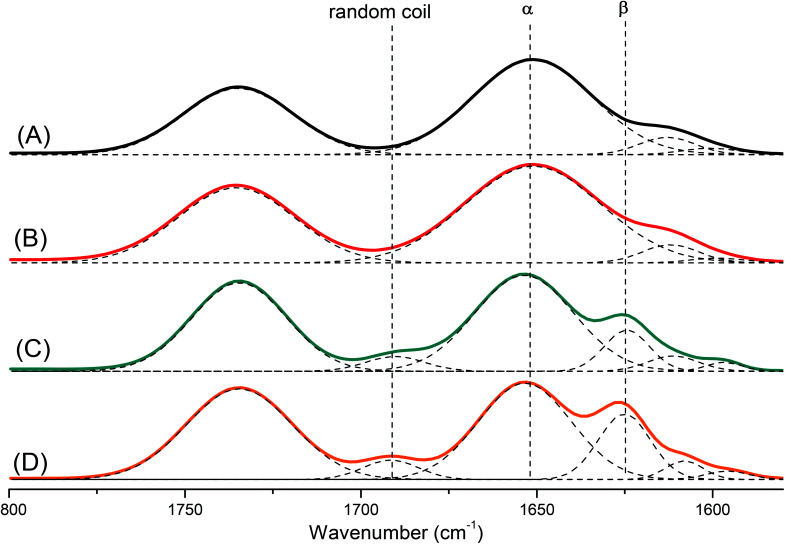
FTIR spectra, recorded at room temperature, displaying the region 1800–1500 cm^−1^, of (A) pyrene-DPA-PBLG(24), (B) pyrene-DPA-PBLG(19), (C) pyrene-DPA-PBLG(9), and (D) pyrene-DPA-PBLG(6).

Polypeptides are attractive materials because they can form various secondary structures. It has reported recently that the degree of polymerization strongly affects the secondary structure of a polypeptide.^47^ To study this behavior more deeply, we prepared a series of pyrene-DPA-PBLG polypeptides having degrees of polymerization (DPs) of 6, 9, 19, and 24. We then used FTIR spectroscopy to obtain information about the secondary structure of each prepared polypeptide. As displayed in Fig. 5, we analyzed the FTIR spectra using the second-derivative technique,^48^ which revealed that the α-helical secondary structure of a pyrene-DPA-PBLG polypeptide was characterized by an amide I band at 1651 cm^−1^. For pyrene-DPA-PBLG polypeptides possessing a β-sheet secondary structure, the amide I band appeared at 1624 cm^−1^, in addition to a band located at 1691 cm^−1^ representing the random coil structure. The free C=O groups in the side chains of pyrene-DPA-PBLG were represented by a signal at 1735 cm^−1^. The quantities of the α-helical, β-sheet, and random coil structures in the pyrene-DPA-PBLG polypeptides were calculated using the deconvolution technique, with a series of Gaussian distributions fitted to each amide I region; Table 2 summarizes the results.

**Table 2:** Fractions of α, β and random coil in pyrene-DPA-PBLG from the analysis of FTIR spectra

Polypeptide	α-helical fraction^a^ (%)	β-sheet fraction^a^ (%)	Random coil fraction^a^ (%)
Pyrene-DPA-PBLG(24)	100	—	—
Pyrene-DPA-PBLG(19)	96.6	—	3.4
Pyrene-DPA-PBLG(9)	65.1	22.5	12.4
Pyrene-DPA-PBLG(6)	49.9	33.7	16.2

a Determined from the curve-fitting of FTIR data in the region from 1800–1550 cm^−1^.

Papadopoulos *et al.* reported that PBLG with a low DP (<18) was present as a mixture of α-helical and β-sheet structures, while the α-helical secondary structure was favored at a high DP (>18).^49^ Similarly, we found that pyrene-DPA-PBLG polypeptides with DPs of 24 and 19 existed mainly as α-helical structures (100 and 96.6%, respectively). In contrast, both secondary structures were observed for pyrene-DPA-PBLG peptides having DPs of 9 and 6 (Fig. 5, Table 2). Thus, we could define the secondary structures of the polypeptides by controlling their DPs—potentially useful for a wide range of specific applications.

**Fig. 6:**
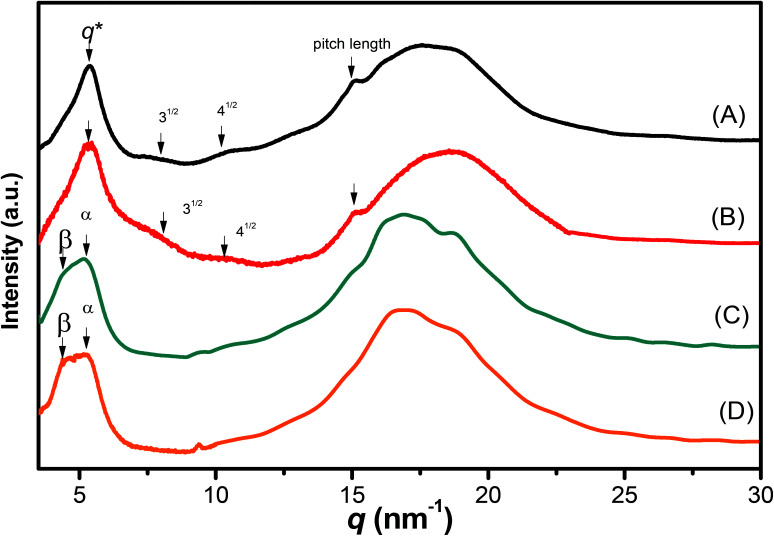
WAXD profiles of (A) pyrene-DPA-PBLG(24), (B) pyrene-DPA-PBLG(19), (C) pyrene-DPA-PBLG(9), and (D) pyrene-DPA-PBLG(6).

We recorded WAXD patterns from the synthesized polypeptides to confirm their secondary structures. As illustrated in Fig. 6, the WAXD patterns of pyrene-DPA-PBLG(24) and pyrene-DPA-PBLG(19) revealed the presence of only α-helical structures, whereas the patterns of pyrene-DPA-PBLG(9) and pyrene-DPA-PBLG(6) revealed both secondary structures, in agreement with the FTIR spectroscopic data. The first strong signal in the diffraction patterns of pyrene-DPA-PBLG(9) and pyrene-DPA-PBLG(6) appeared at a value of *q* of 4.5 nm^−1^, attributable to the distance (*d* = 1.38 nm) between the backbones in the antiparallel β-sheet structure. Another diffraction peak at a value of *q* of 16.8 nm^−1^ (*d* = 0.37 nm) represented the intermolecular distance between neighboring polypeptide backbone chains in one lamella. In the WAXD patterns of pyrene-DPA-PBLG(24) and pyrene-DPA-PBLG(19), the primary diffraction signal at 4.5 nm^−1^, corresponding to the β-sheet secondary structure, had disappeared, with only a strong diffraction signal (q*) appearing at 5.37 nm^−1^, suggesting the absence of β-sheet structures for the longer polypeptides. The primary signal (q*) and two other signals at 7.9 and 10.3 nm^−1^, with relative positions 1 : 3^1/2^ : 4^1/2^, is a typical indication of the formation of an α-helical secondary structure. These three signals represented the (10), (11), and (20) reflections of two-dimensional hexagonally packed cylinders consisting of 18/5 α-helices with a cylinder distance of 1.16 nm.

The FTIR spectra and WAXD patterns provided the following information: for pyrene-DPA-PBLG(9) and pyrene-DPA-PBLG(6), with low average DPs (<19), both α-helical and β-sheet secondary structures were present; when the DP was greater than 19, however, the β-sheet structures disappeared and only α-helical structures were favored.

### Solution UV-Vis and PL emission spectra

We studied the photophysical properties of pyrene-DPA-2NH_2_ and pyrene-DPA-PBLG from their UV-Vis absorption and PL emission spectra recorded at a concentration of 10^−4^ M in various solvents. Pyrene-DPA-2NH_2_ has high solubility in MeOH, THF, DCM, DMF, and acetone; the pyrene-DPA-PBLG(*n*) polypeptides displayed high solubility in MeOH, THF, DMF, and DMSO, but low solubility in DCM and acetone. As illustrated in Fig. S3,† absorption maxima of pyrene-DPA-2NH_2_ and pyrene-DPA-PBLG appeared in the regions 290–350 and 275–350 nm, representing the n–π* and π–π transitions of the conjugated pyrenyl and phenyl segments. In addition, an absorption band appeared in the region 375–500 nm for both pyrene-DPA-2NH_2_ and pyrene-DPA-PBLG, arising from dimerization of the pyrene segments. Moreover, a strong solvent effect was evident in the absorption spectra of pyrene-DPA-2NH_2_ [Fig. S3(A)†]. The absorbance maximum of pyrene-DPA-2NH_2_ in THF, DCM, and DMF appeared at 313 nm; in acetone, the absorption maximum red-shifted to 326 nm, due to the strong guest–host interactions between pyrene-DPA-2NH_2_ and the acetone environment. In contrast, the absorption maximum of pyrene-DPA-2NH_2_ blue-shifted to 306 nm in MeOH because of the strong hydrogen bonding between the fluorophore and MeOH molecules, thereby increasing the stability of pyrene-DPA-2NH_2_ in the ground state. Interestingly, we observed that the intensity of the absorbance band at 427 nm of pyrene-DPA-PBLG in DMF increased upon decreasing the DP [Fig. S3(B)†]: DPs of 24, 19, 9, and 6 provided absorbance intensities of 0.25, 0.44, 0.65, and 0.83 au, respectively. This behavior suggests that dimerization of the pyrene units in the short-chain polypeptides occurred more readily than that in the long-chain polypeptides.

**Fig. 7:**
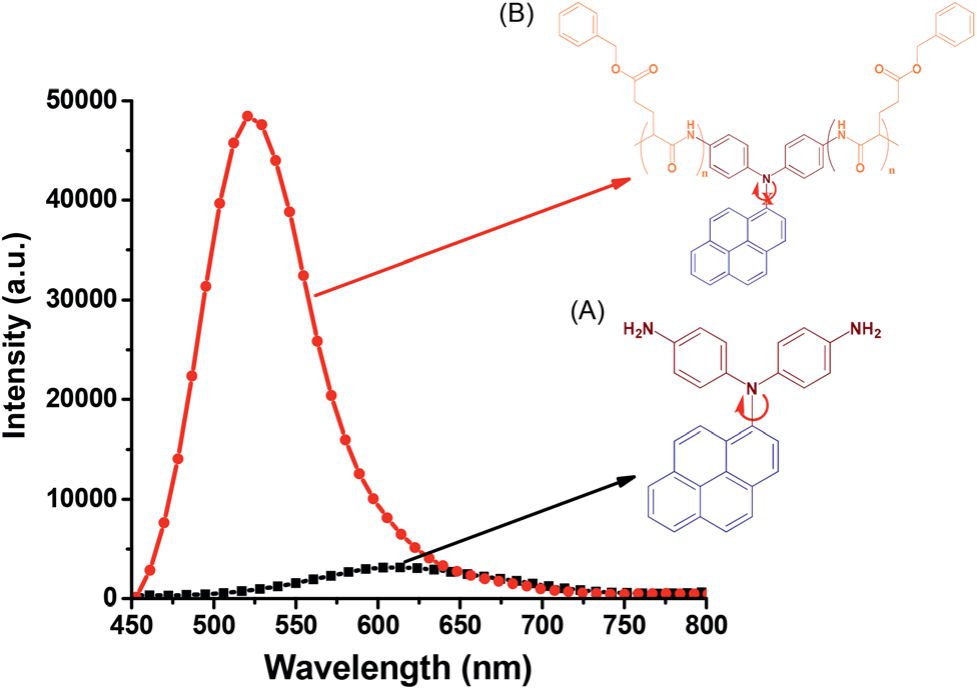
Solution PL emission spectra of (A) pyrene-DPA-2NH_2_ and (B) pyrene-DPA-PBLG(6) in THF (concentration: 10^−3^ M; excitation wavelength: 343 nm).

**Fig. 8:**
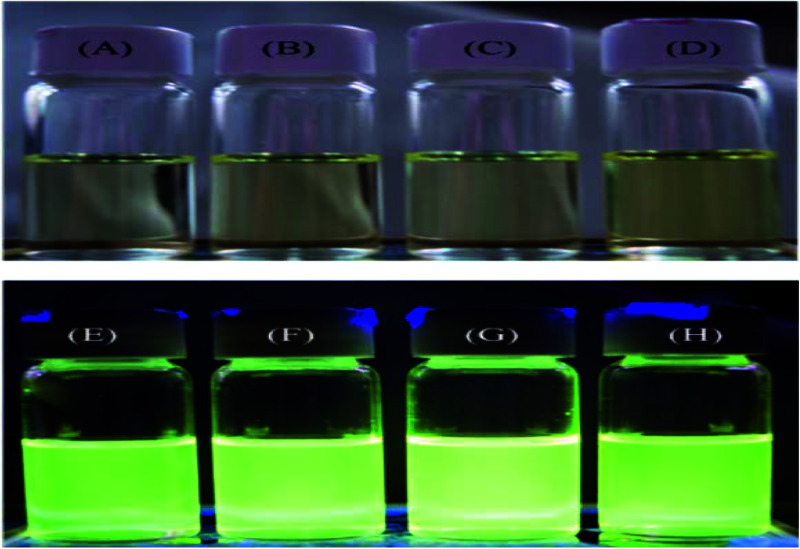
PL images of (A and E) pyrene-DPA-PBLG(24), (B and F) pyrene-DPA-PBLG(19), (C and G) pyrene-DPA-PBLG(9), and (D and H) pyrene-DPA-PBLG(6) in THF (concentration: 10^−3^ M) (A–D) before and (E–H) after UV irradiation at 343 nm.

In contrast to the absorption spectra, pyrene-DPA-2NH_2_ exhibited almost no fluorescence emission in MeOH, THF, DMF, or acetone, and little-improved emission in DCM, while all of the pyrene-DPA-PBLG polypeptides displayed strong fluorescence emissions in MeOH, THF, DMF, and DMSO. For example, pyrene-DPA-2NH_2_ has weak emission in DCM at a concentration of 10^−3^ M, while pyrene-DPA-PBLG(5) had a 16-fold stronger emission in THF at the same concentration (Fig. 7). This behavior is consistent with pyrene-DPA-2NH_2_ possessing a flexible DPA moiety, which can undergo dynamic intramolecular rotation (IR), thereby quickly quenching its excited states and resulting in the absence of luminescence (Fig. 7). Conversely, the strong emissions of the pyrene-DPA-PBLG polypeptides were due to the intramolecular rotations being restricted by the PBLG side chains. The blue shift of pyrene-DPA-PBLG(6) in comparison with pyrene-DPA-2NH_2_ could be attributed to the hydrogen bond interaction between the oxygen atom of THF solvent and the N–H group of the polypeptide, which increased the stability of pyrene-DPA-PBLG(6) in the ground state. Additionally, this blue shift can also arise from the conversion of pyrene excimer from dynamic excimer within pyrene-DPA-2NH_2_ into static excimer within pyrene-DPA-PBLG(6). As previously reported, pyrenes can be formed two kinds of excimers; dynamic and static excimers.^50^ The first one occurred when an excited-state molecule of pyrene formed a dimer with another ground-state molecule of pyrene, while the second one occurred when a pyrene dimer formed firstly in the ground state and then excited. Where, the hydrogen bonding between pyrene-DPA-PBLG(6) polypeptide chains induced pyrene molecules to come in close proximity to each other and form a dimer in the ground state. The images of the pyrene-DPA-PBLG polypeptides in Fig. 8 indicate that the fluorescence color of a THF solution of each of these polypeptides was greenish yellow. This paper is, therefore, the first to report the turning-on of the radiative pathway of a non-fluorescent molecule in the presence of PBLG chains, providing strongly emissive polypeptides; this approach could be used to tailor fluorophore-containing polypeptides with varying functionalities for specific applications. Therefore, we performed further experiments to study the fluorescence behavior of these kinds of polypeptides.

**Fig. 9:**
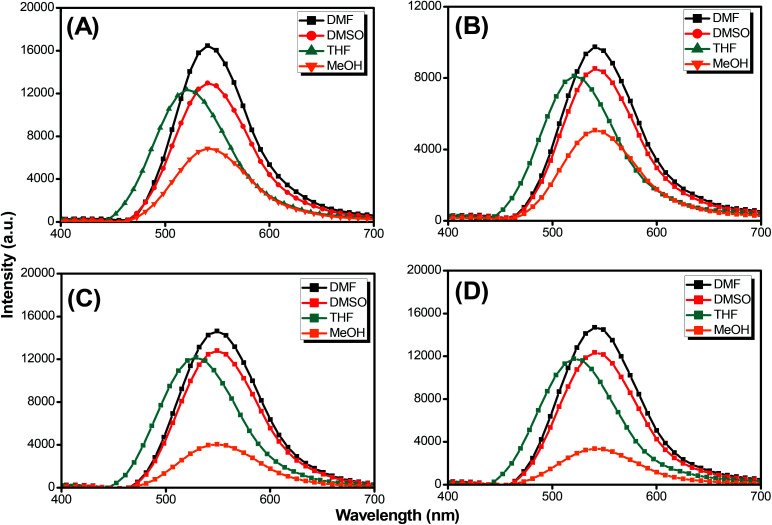
Solution PL emission spectra of (A) pyrene-DPA-PBLG(24), (B) pyrene-DPA-PBLG(19), (C) pyrene-DPA-PBLG(9), and (D) pyrene-DPA-PBLG(6) in various solvents (concentration: 10^−4^ M; excitation wavelength: 343 nm).

Pyrene compounds exhibit environmental solvatochromic effects, in which the relative maximum of the emission bands is strongly dependent on the solvent polarity.^51^ Therefore, to study the solvatochromic behavior of our synthesized pyrene-DPA-PBLG polypeptides, we investigated their fluorescence emissions in solvents of various polarities in which they could be dissolved, namely THF, MeOH, DMF, and DMSO. As illustrated in Fig. 9, the pyrene-DPA-PBLG polypeptides exhibited fluorescence emission peaks at 542, 540, and 541 nm in MeOH, DMF, and DMSO, respectively. These emission peaks arose from π–π* transitions in the pyrenyl and phenyl units. The emissions of the pyrene-DPA-PBLG polypeptides underwent hypsochromic shifts upon decreasing the solvent polarity; for example, the emission peaks of the polypeptides appeared at 519 nm in THF. We attribute this solvatochromic behavior to rapid amine-to-pyrene and amine-to-amide intramolecular charge-transfer (ICT) processes in the excited state, as well as the stability of the excited states in the high-polarity solvents.^52^ In particular, the emission maximum of the pyrene-DPA-PBLG polypeptides was dependent upon the solvent polarity (*i.e.*, solvatochromic behaviour). This behavior is typical of pyrene derivatives and TPA-containing compounds.^53^ Moreover, as shown in Fig. 9A–D, all pyrene-DPA-PBLG(*n*) polypeptides with different degrees of polymerization have the same the solvatochromic behavior in solutions which indicated that the α-helical and β-sheet secondary structures of our polypeptides did not affect the fluorescence properties of polypeptides.

### Aggregation-induced emission (AIE)

**Fig. 10:**
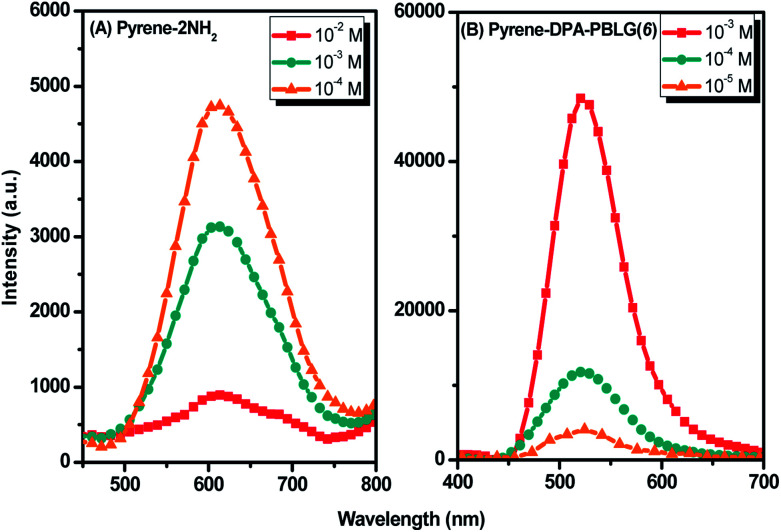
Solution PL emission spectra of (A) pyrene-DPA-2NH_2_ in DCM and (B) Pyrene-DPA-PBLG(6) in THF (excitation wavelength: 343 nm).

Luminescent materials having strong π-conjugated systems typically exhibit a strong emission in diluted solutions; in concentrated solutions or in the aggregate (solid) state, however, their fluorescence emissions can be very weak. This ACQ behavior arises as a result of noncovalent intermolecular interactions (*e.g.*, π-stacking).^54^ To examine the AIE behavior of pyrene-DPA-2NH_2_ and pyrene-DPA-PBLG(*n*), we evaluated their solution PL behavior using the concentration effect and solvent/nonsolvent pairs. We investigated the concentration effect by measuring the fluorescence emissions of pyrene-DPA-2NH_2_ and pyrene-DPA-PBLG(6) at various concentrations in DCM and THF, respectively. As displayed in Fig. 10(A), the emission intensity of pyrene-DPA-2NH_2_ decreased upon increasing the solution concentration. This behavior, termed “concentration-quenched emission,” arose from strong face-to-face π-stacking interactions of neighboring pyrene units, leading to the formation of excimers, which were quickly quenched. Conversely, upon increasing the concentration of pyrene-DPA-PBLG(*n*) in solution from 10^−5^ to 10^−3^ M, the intensity of the emission increased gradually [Fig. 10(B) and S4†]. This behavior, termed “concentration-enhanced emission,” can be ascribed to the AIE effect. On the whole, the concentration effect strongly suggested that the PL emission of pyrene-DPA-2NH_2_ transformed from ACQ to AIE after incorporation into the main chain of PBLG, and formed an AIE-active polypeptide. As previously reported, the common method to prepare AIE-active polypeptide^55^ or polymer^56^ is depended on polymerization reaction in which AIE materials were utilized as initiators. On the contrary, in this study, we prepared AIE-active polypeptides through the ROP of BLG-NCA initiated with an ACQ material (pyrene-DPA-2NH_2_).

**Fig. 11:**
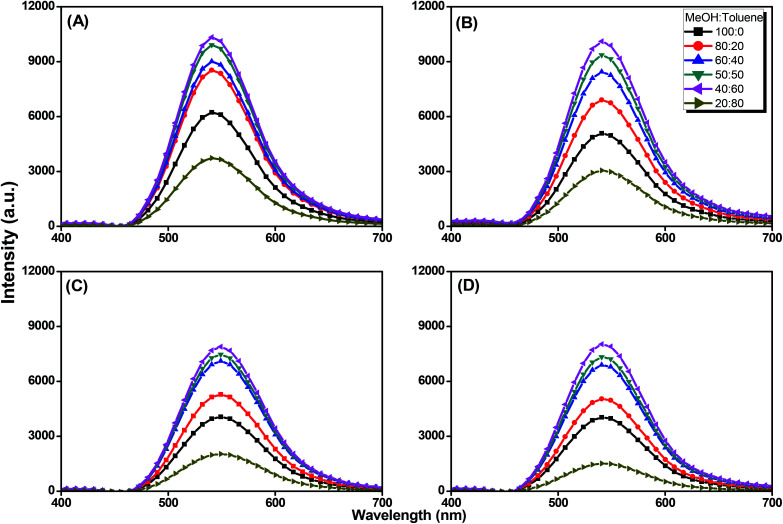
Solution PL emission spectra of (A) pyrene-DPA-PBLG(24), (B) pyrene-DPA-PBLG(19), (C) pyrene-DPA-PBLG(9), and (D) pyrene-DPA-PBLG(6) in the MeOH/toluene (concentration: 10^−4^ M; excitation wavelength: 343 nm).

**Fig. 12:**
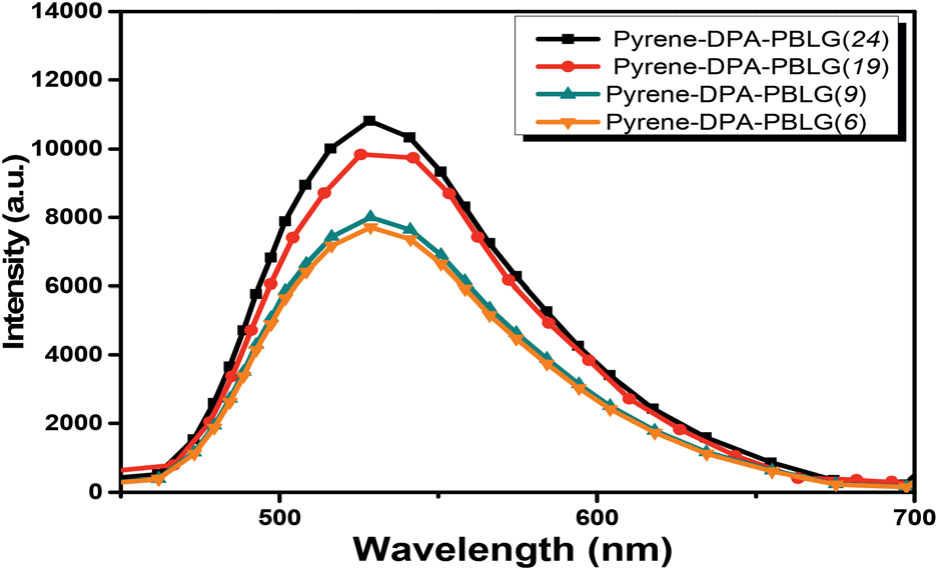
PL emission spectra of pyrene-DPA-PBLG(*n*) in the solid state (excitation wavelength: 343 nm).

We further evaluated the AIE behavior of the pyrene-DPA-PBLG polypeptides by measuring their PL emissions in solvent/nonsolvent pairs (MeOH/toluene mixtures; Fig. 11). We selected MeOH as the solvent and toluene as the poor solvent, gradually aggregating the polypeptides by increasing the concentration of toluene. As displayed in Fig. 11, all of the diluted (10^−4^ M) solutions of pyrene-DPA-PBLG(*n*) in MeOH exhibited weak PL emissions. Interestingly, the resultant intensity of the fluorescence emission increased continuously upon increasing the concentration of toluene from 20 to 60 vol%, but decreased suddenly for each of the solutions at 80 vol% toluene. The PL emissions were enhanced in the solutions upon increasing the toluene content from 20 to 60 vol% because of aggregation, consistent with AIE behavior. We attribute the unexpected decrease in emission for the 80 vol% toluene solutions to precipitation of pyrene-DPA-PBLG(*n*) in solutions consisting mainly of the nonsolvent toluene. The precipitation of polypeptide after increasing the nonsolvent content has been reported by Hong *et al.*^57^ Consequently, the formed precipitates were isolated by centrifuging and then investigated by FTIR, which found to be pyrene-DPA-PBLG(*n*). Further, the fluorescence of pyrene-DPA-PBLG(*n*) in the solid state was also investigated, as shown in Fig. 12. Pyrene-DPA-PBLG(*n*) polypeptides showed a massive PL emission peak at 528 nm which confirmed the AIE behavior of our polypeptides. These PL data confirmed that all of the pyrene-DPA-PBLG polypeptides exhibited AIE behavior, whereas pyrene-DPA-2NH_2_ displayed ACQ behavior. Additionally, in comparison with PL data in Fig. 11(A)–(D) and 12, we can confirm that the pyrene-DPA-PBLG(24) and pyrene-DPA-PBLG(19) with only α-helical secondary structure and pyrene-DPA-PBLG(9) and pyrene-DPA-PBLG(6) with both α-helical and β-sheet secondary structures are AIE materials. Thus, the AIE behaviour of these polypeptides was not dependent upon the changing of secondary structure content.

### Pyrene-DPA-PBLG/MWCNTs composites: fabrication and CNT dispersion

**Fig. 13:**
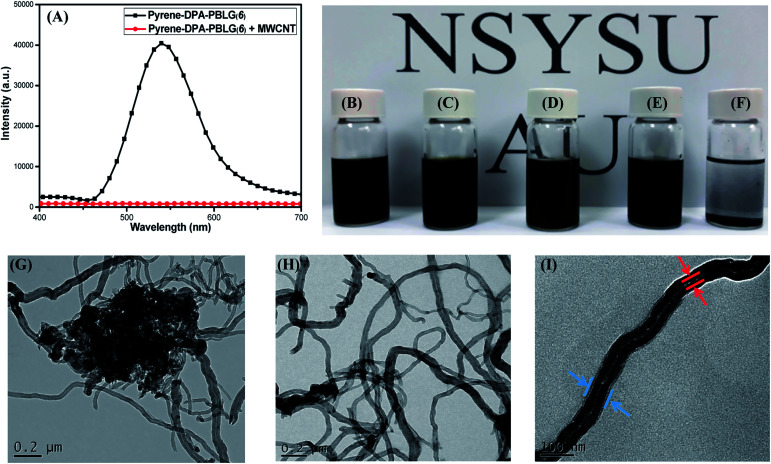
(A) PL emission spectra of pyrene-DPA-PBLG(6) and pyrene-DPA-PBLG(6)/MWCNT. (B–F) Photographs of DMF dispersions of (B) pyrene-DPA-PBLG(24)/MWCNT, (C) pyrene-DPA-PBLG(19)/MWCNT, (D) pyrene-DPA-PBLG(9)/MWCNT, (E) pyrene-DPA-PBLG(6)/MWCNT, and (F) pristine MWCNTs. (G–I) TEM images of (G) pure MWCNTs after sonication in DMF and (H and I) pyrene-DPA-PBLG(19)/MWCNT dispersions on (H) large and (I) small scales.

Because of strong π-stacking between CNTs, the dispersion of MWCNTs in solvents can be difficult. Many supramolecular complexes have, however, been reported as dispersing agents for MWCNTs, due to their beneficial noncovalent interactions.^58^ Polypeptides themselves are feeble dispersing agent for MWCNTs because the interactions between these species are weak. Nevertheless, we suspected that the incorporation of components (*e.g.*, pyrene units) capable of strong π-stacking might increase the interactions between polypeptides and MWCNTs and, thus, increase the dispersion of MWCNTs. Accordingly, we examined our pyrene-DPA-PBLG polypeptides, which feature pyrene units in their backbones, as dispersing agents for MWCNTs. Fig. 13(A) displays the PL emission spectra of pyrene-DPA-PBLG(6) (chosen as an example polypeptide) and the pyrene-DPA-PBLG(6)/MWCNT complex in DMF solution after excitation at 343 nm. Pyrene-DPA-PBLG(6) exhibited a strong PL emission at 540 nm, reflecting the presence of the pyrene unit, while the pyrene-DPA-PBLG(6)/MWCNT complex displayed very weak PL emission. This behavior suggests that the pyrene units interacted strongly with the MWCNTs through π-stacking, leading to energy transfer from the light-emitted pyrene units to the MWCNTs and then quenching of the polypeptide's fluorescence.^59^ The presence of interactions between the pyrene-DPA-PBLG(*n*) polypeptides and the MWCNTs is further evident in Fig. 13(B)–(F), which display photographs of the pyrene-DPA-PBLG(24)/MWCNT, pyrene-DPA-PBLG(19)/MWCNT, pyrene-DPA-PBLG(9)/MWCNT, and pyrene-DPA-PBLG(6)/MWCNT complex dispersions and pristine MWCNT dispersion, respectively, in DMF after 24 h. As shown in Fig. 13(F), the pristine MWCNTs revealed a high degree of aggregation after 24 h, which compatible with the reported finding.^60^ In contrast, none of the pyrene-DPA-PBLG/MWCNT complex dispersions exhibited any obvious aggregation [Fig. 13(B)–(E)]. Moreover, TEM imaging analysis confirmed the presence of stabilizing interactions between our synthesized polypeptides and the MWCNTs. Fig. 13(G)–(I) reveal that the pristine MWCNTs underwent a high degree of aggregation in DMF, due to strong π–π interactions, whereas the pyrene-DPA-PBLG(19)/MWCNT complexes formed uniform dispersions of aggregated MWCNTs, stabilized through a series of noncovalent interactions. In brief, we conclude that our pyrene-DPA-PBLG polypeptides have the ability to disperse MWCNTs effectively—a property that might be useful for medicinal and manufacturing applications.

Next, we examined the thermal stabilities of the pyrene-DPA-PBLG/MWCNTs composites; all of the pyrene-DPA-PBLG/MWCNT composite dispersions in DMF were dried under vacuum to remove the solvent and then TGA was performed from 40 to 800 °C under N_2_ at a heating rate of 10 °C min^−1^ (Fig. S5†). We used the char yield at 600 °C as the standard. Pyrene-DPA-PBLG(24), pyrene-DPA-PBLG(19), pyrene-DPA-PBLG(9), and pyrene-DPA-PBLG(6) provided char yields of 14.0, 16.6, 22.2, and 22.3%, respectively. In contrast, the pyrene-DPA-PBLG(24)/MWCNT, pyrene-DPA-PBLG(19)/MWCNT, pyrene-DPA-PBLG(9)/MWCNT, and pyrene-DPA-PBLG(6)/MWCNT composites provided char yields of 79.3, 79.4, 91.0, and 91.1%, respectively. According to the char yield of the pristine MWCNTs (95.9% at 600 °C), we estimated the contents of pure pyrene-DPA-PBLG(*n*) polypeptides on MWCNTs and found to be 166, 165, 49 and 48 mg g^−1^ for pyrene-DPA-PBLG(24)/MWCNT, pyrene-DPA-PBLG(19)/MWCNT, pyrene-DPA-PBLG(9)/MWCNT, and pyrene-DPA-PBLG(6)/MWCNT composites, respectively. These results suggest that the interactions of the α-helical structures of our polypeptides with the MWCNTs were much stronger than those of β-sheets, possibly because the long helical-chains in the α-helical structures increased the face-to-face interactions of the pyrene units with the MWCNTs.

## Conclusion

We have used a simple ROP to prepare a series of new polypeptides, each containing a di(4-aminophenyl)pyrenylamine luminophore, with various DPs. The chemical structures and DPs of the polypeptides were confirmed using FTIR, NMR, and MALDI-TOF spectroscopy. The polypeptides with DPs of less than 19 were mixtures of α-helical and β-sheet structures, while those with higher DPs featured only α-helical structures. Interestingly, PL data revealed that pyrene-DPA-2NH_2_ has a weak fluorescence emission, due to the strong IRs, which quickly quenched its excited states, while the emissions of the pyrene-DPA-PBLG polypeptides were 16-fold stronger, due to restriction of the IRs induced by the presence of the PBLG side chains. In addition, pyrene-DPA-2NH_2_ is an ACQ compound, but it became a strongly AIE compound after incorporation into the PBLG segments with rigid-rod conformations. Furthermore, pyrene-DPA-PBLG/MWCNT composites were highly dispersible in DMF as a result of noncovalent interactions between the pyrene units in the polypeptides and the MWCNT. Fluorescence spectroscopy and photographic and TEM images confirmed the dispersibility of our polypeptides and their π–π interactions with the MWCNTs. The development of such multifunctional polypeptides displaying AIE behavior and π–π interactions may lead to wider biomedical applicability. In addition, our new pyrene-DPA-PBLG/MWCNT could be used as carbon nanotube/fluorescent peptide probe for sensitive detection of enzyme activity such as cancer-related enzymes.

## Conflicts of interest

There are no conflicts to declare.

## Supplementary Material

RA-008-C8RA02369G-s001
