# Supplementary material for: Diphenylpyrenylamine-functionalized polypeptides: secondary structures, aggregation-induced emission, and carbon nanotube dispersibility
Source: RSC Adv. 2018 Apr 23;8(28):15266–81. doi: 10.1039/c8ra02369g (PMC9080069; doi:10.1039/c8ra02369g)
Supplement: RA-008-C8RA02369G-s001 [file RA-008-C8RA02369G-s001.pdf]

Electronic Supplementary Material (ESI) for RSC Advances.
This journal is © The Royal Society of Chemistry 2018

Supporting Information for

Diphenylpyrenylamine-Functionalized Polypeptides: Secondary Structures, Aggregation-Induced Emission, and Carbon Nanotube Dispersibility

Ahmed F. M. EL-Mahdy[a,b] and Shiao-Wei Kuo[a]

[a]Department of Materials and Optoelectronic Science, National Sun Yat-Sen University, Kaohsiung 80424, Taiwan

[b]Chemistry Department, Faculty of Science, Assiut University, Assiut 71516, Egypt.

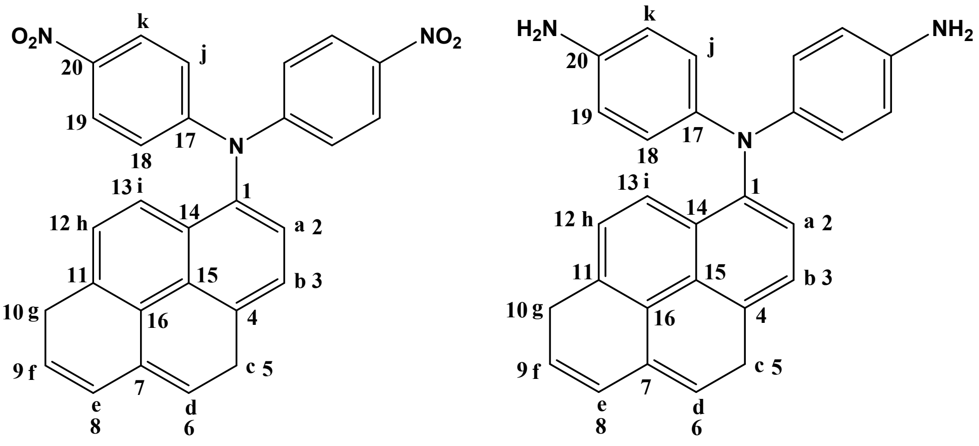

(a) (b)

Scheme S1. NMR spectral peak assignments of (a) pyrene-DPA-2NO$_2$ and (b) pyrene-DPA-2NH$_2$.

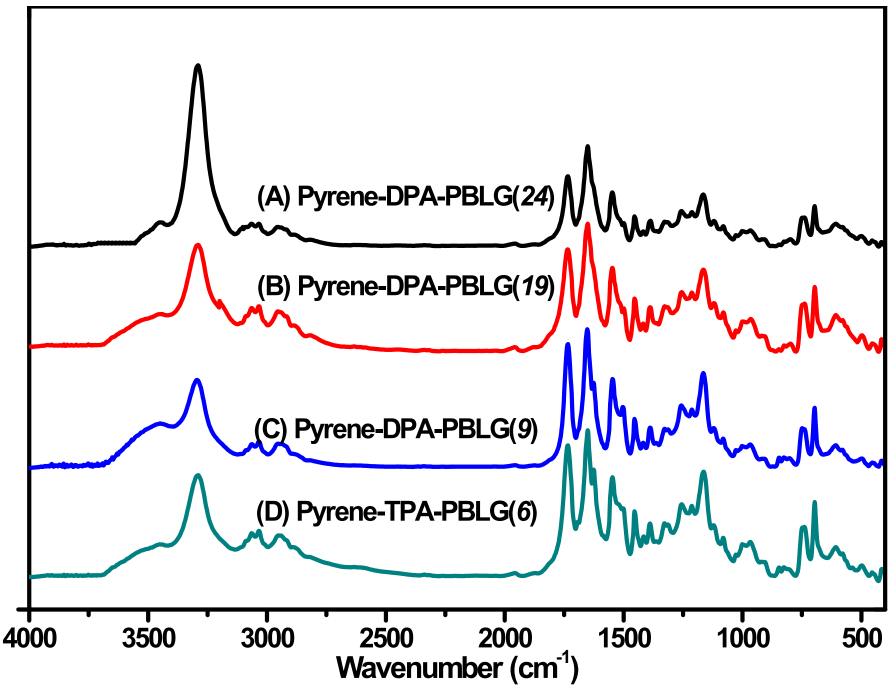

Figure S1. FTIR spectra of (A) pyrene-DPA-PBLG(*24*), (B) pyrene-DPA-PBLG(*19*), (C) pyrene-DPA-PBLG(*9*), and (D) pyrene-DPA-PBLG(*6*), recorded at room temperature.

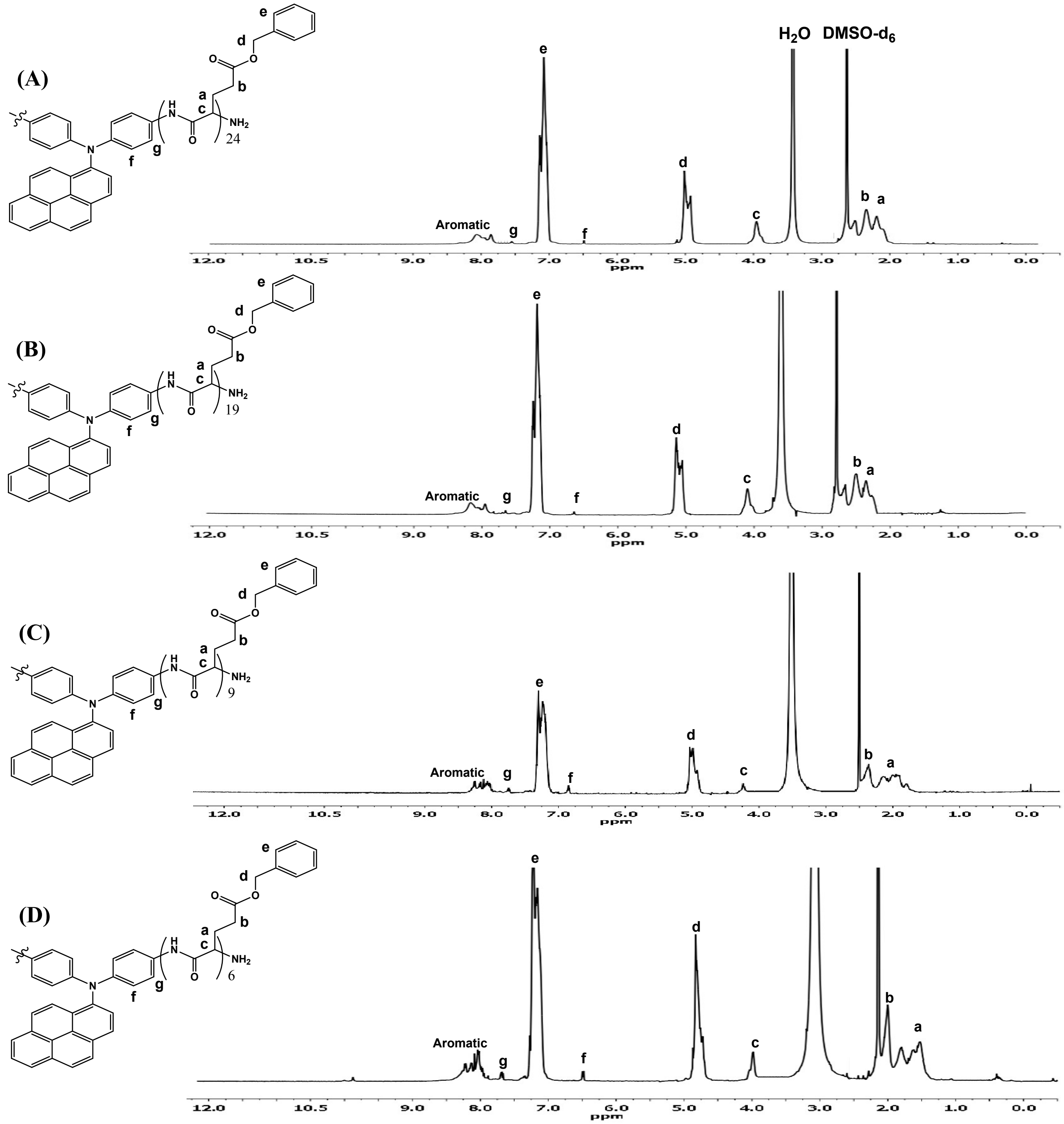

Figure S2. ^{1}H NMR spectra of (A) pyrene-DPA-PBLG(*24*), (B) pyrene-DPA-PBLG(*19*), (C) pyrene-DPA-PBLG(*9*), and (D) pyrene-DPA-PBLG(*6*).

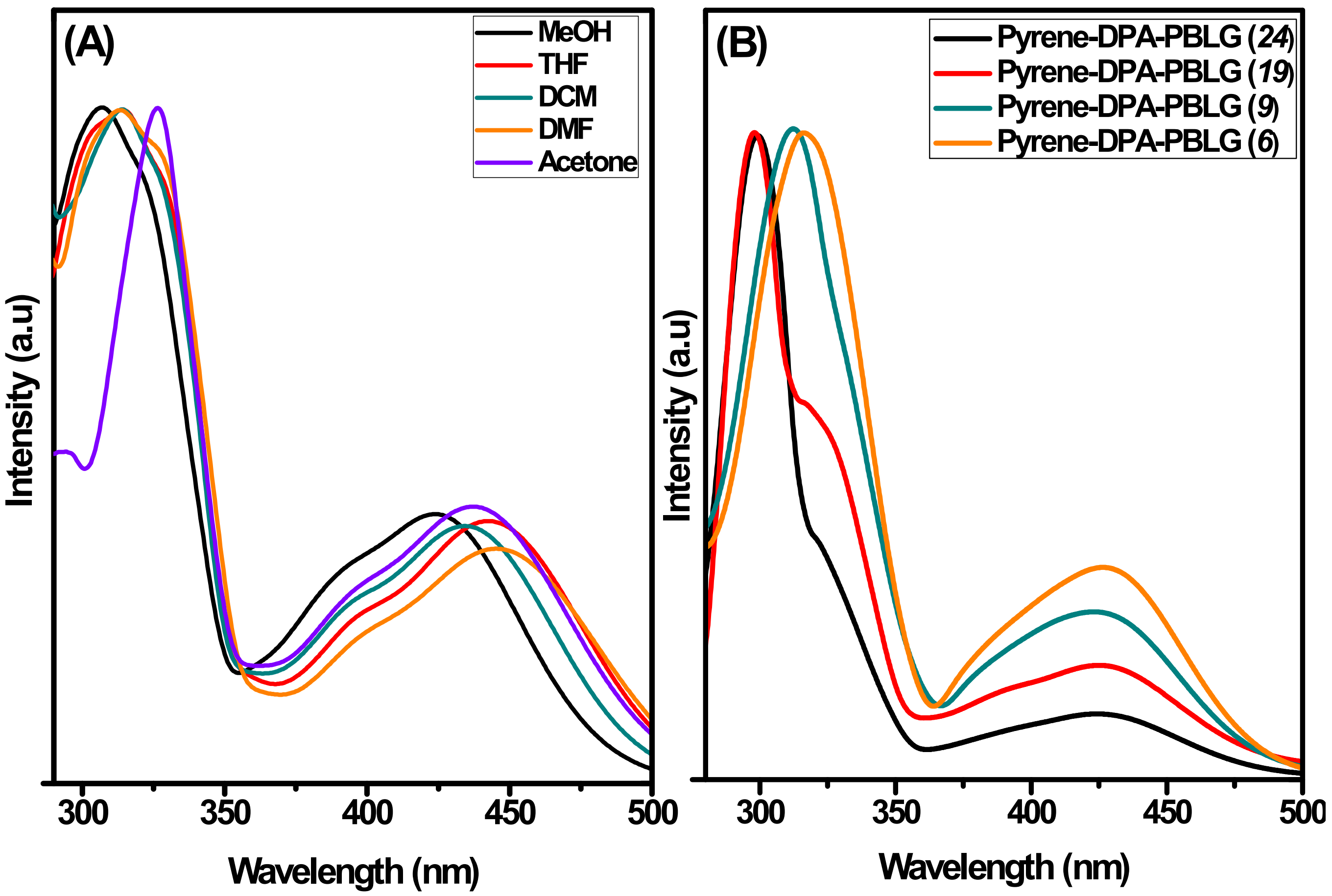

Figure S3. UV–Vis absorption spectra of (A) pyrene-DPA-2NH_2 and (B) pyrene-DPA-PBLG in DMF (concentration: 10^{-4} M).

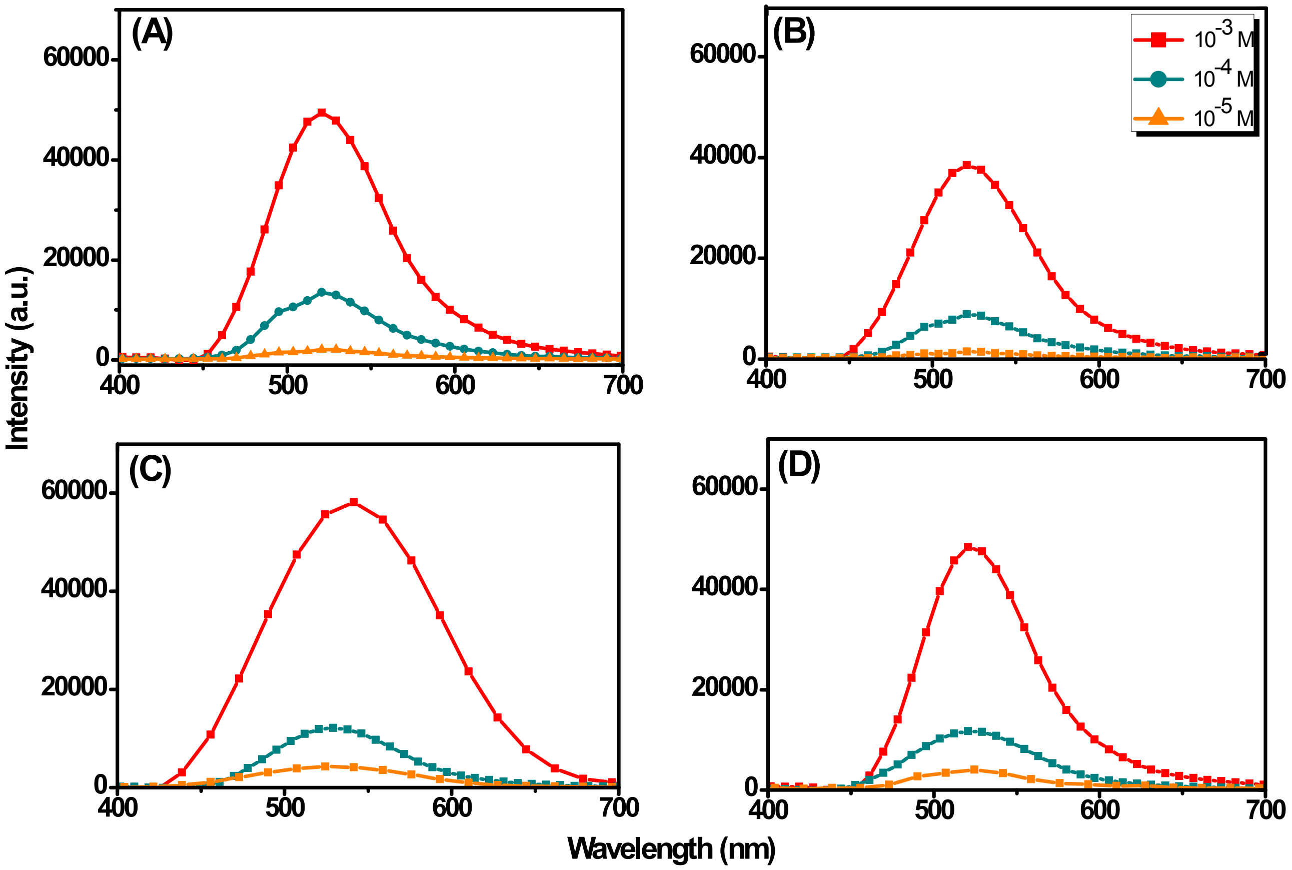

Figure S4. Solution PL emission spectra of (A) pyrene-DPA-PBLG(*24*), (B) pyrene-DPA-PBLG(*19*), (C) pyrene-DPA-PBLG(*9*), and (D) pyrene-DPA-PBLG(*6*) in THF (excitation wavelength: 343 nm).

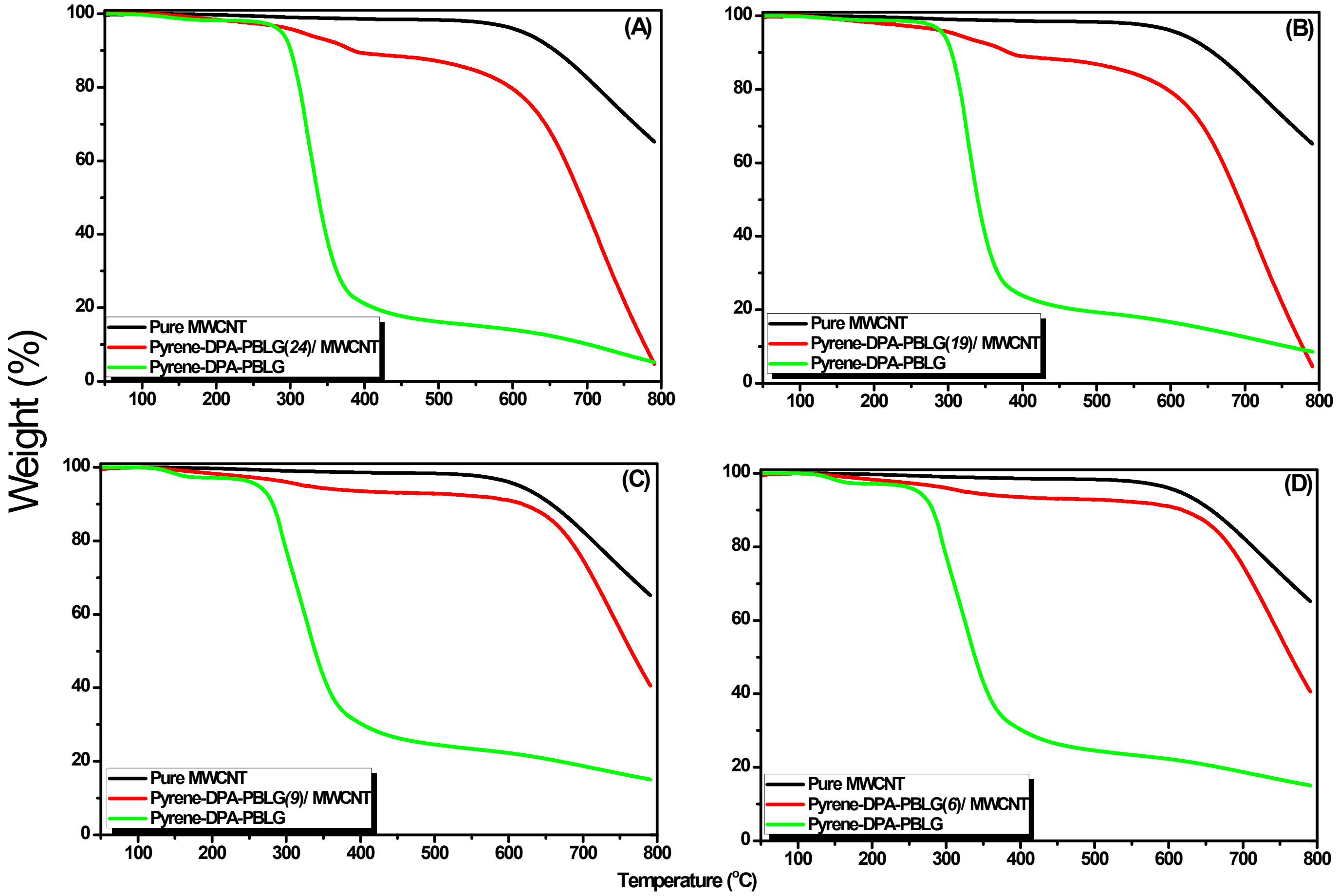

Figure S5. TGA analyses of (A) pyrene-DPA-PBLG(*24*), (B) pyrene-DPA-PBLG(*19*), (C) pyrene-DPA-PBLG(*9*), and (D) pyrene-DPA-PBLG(*6*) as composites with MWCNTs.
